# Supervised machine learning for automated classification of human Wharton’s Jelly cells and mechanosensory hair cells

**DOI:** 10.1371/journal.pone.0245234

**Published:** 2021-01-08

**Authors:** Abihith Kothapalli, Hinrich Staecker, Adam J. Mellott

**Affiliations:** 1 Blue Valley West High School, Overland Park, KS, United States of America; 2 Department of Otolaryngology, Head and Neck Surgery, University of Kansas Medical Center, Kansas City, KS, United States of America; 3 Department of Plastic Surgery, University of Kansas Medical Center, Kansas City, KS, United States of America; Torrens University Australia, AUSTRALIA

## Abstract

Tissue engineering and gene therapy strategies offer new ways to repair permanent damage to mechanosensory hair cells (MHCs) by differentiating human Wharton’s Jelly cells (HWJCs). Conventionally, these strategies require the classification of each cell as differentiated or undifferentiated. Automated classification tools, however, may serve as a novel method to rapidly classify these cells. In this paper, images from previous work, where HWJCs were differentiated into MHC-like cells, were examined. Various cell features were extracted from these images, and those which were pertinent to classification were identified. Different machine learning models were then developed, some using all extracted data and some using only certain features. To evaluate model performance, the area under the curve (AUC) of the receiver operating characteristic curve was primarily used. This paper found that limiting algorithms to certain features consistently improved performance. The top performing model, a voting classifier model consisting of two logistic regressions, a support vector machine, and a random forest classifier, obtained an AUC of 0.9638. Ultimately, this paper illustrates the viability of a novel machine learning pipeline to automate the classification of undifferentiated and differentiated cells. In the future, this research could aid in automated strategies that determine the viability of MHC-like cells after differentiation.

## 1. Introduction

Damage to cochlear mechanosensory hair cells (MHCs) can lead to permanent hearing impairment known as sensorineural hearing loss (SNHL) [1]. SNHL affects over ten percent of the population [2] and arises from several etiologies, including prolonged exposure to loud noises [3], certain antibiotics [4], and acoustic neuroma [5]. Tissue engineering and gene therapy are two promising strategies that endeavor to reverse this damage and repair these tissues [6,7].

Tissue engineering strategies utilize stem cells to regenerate damaged tissues by seeding progenitor cells or stem cells into biomaterial scaffolds [8]. This process is used to direct stem cells toward a specific terminal lineage. In the case of cochlear damage, tissue engineering strategies are used to differentiate stem cells toward a cochlear MHC phenotype, creating MHC-like cells. Another method to repair MHCs involves gene therapy, whereby mesenchymal stem cells, such as human Wharton’s Jelly cells (HWJCs), are transfected with nucleic acids that either increase or inhibit expression of specific genes [9]. Genetic reprogramming results in the differentiation of HWJCs, similarly creating MHC-like cells [10,11]. Both of these strategies, however, do not reach one hundred percent efficiency. Upon the conclusion of these processes, some HWJCs will differentiate into MHC-like cells, while others will retain a HWJC phenotype or an intermediate phenotype [12,13]. Therefore, these methods require scientists to manually inspect samples of cells to determine which cells have successfully differentiated into MHC-like cells before applying them to repair cochlear damage. Identifying differentiated cells is a time-consuming and tedious process, where the scientist must classify hundreds, if not thousands, of cells to determine which are viable.

Machine learning (ML) may present an efficient method to automate cell classification for down-stream analysis and validation of phenotype in tissue engineering or gene therapy settings [14]. ML algorithms are used to detect underlying patterns among large data sets without a predefined model [15]. ML has become widely used in medical image analysis and tissue specimen analysis to classify cell morphologies from cell images [14,16,17]. In several similar works, ML has been able to successfully distinguish between cancerous and healthy cells or determine the severity of a cancerous lesion [18–20]. In the field of tissue engineering specifically, ML has already been used to develop scaffold designs and classify specific tissue constructs [21,22]; however, the potential of ML to speed up the process of cell classification during tissue engineering or gene therapy has yet to be thoroughly investigated.

Given the distinct change in cell morphology between HWJCs and MHCs, ML may pose an accurate and feasible method to distinguish between these two cell phenotypes using size, shape, and texture features computed from phases contrast images of the cells. This method would allow scientists to classify these cells using optical features without the need for immunolabeling or other function testing in the medical world.

Six different ML algorithms are presented in this paper: *L*_*1*_ and *L*_*2*_ regularized logistic regressions (LRs) [23], support vector machine (SVM) [24], K-nearest neighbors (KNN) classifier [25], random forest classifier [26], and multi-layer perceptron (MLP) [27]. These algorithms are among the most commonly used algorithms for supervised learning applications, especially for classification using a quantitative, structured data set, such as the one used in this paper [28,29].

A voting classifier (VC) model is also presented in this paper. This model functions as an ensemble of distinct, individual models that uses the output predictions of the separate models and averages them to obtain one final prediction [30]. The VC model, therefore, has the ability to adjust for individual errors among constituent algorithms, as any error in one model can be counterbalanced by the other models [31].

While deep learning methods, namely convolutional neural networks, may also be viable for the cell classification problem presented in this paper, these methods require greater computational resources and have greater hardware requirements when compared to conventional machine learning algorithms [32]. Furthermore, deep learning methods often require larger data sets to train an efficient network, whereas conventional machine learning algorithms can be sufficiently trained with few data [33]. Thus, the objective of this paper is to demonstrate the viability of a novel computational pipeline for the automated cell segmentation, feature extraction, and classification of HWJCs and MHC-like cells. This pipeline was developed with the intention of achieving similar performance to deep learning methods while requiring fewer computational resources and data for its implementation; moreover, the proposed pipeline could feasibly be adapted to similar cell classification problems without the need for extensive technical expertise or substantial retraining.

## 2. Materials and methods

To determine which ML model is most appropriate for the classification of HWJCs and MHC-like cells, six different ML models were developed and then evaluated. Prior to the development of these models, however, various methods were used for feature extraction from cells within phase contrast images of previously published work. Once the data set was extracted from these images, active features were also identified. The ML algorithms were then implemented using this data set. Each algorithm was developed once using all features in the data set and once using only the active features. The performance under these two conditions was then compared for each algorithm to determine the implications of limiting the algorithms to the active features. The performance across all models was also compared to determine which algorithm had the highest predictive ability. Feature extraction was done using Fiji [34,35] and CellProfiler [36–38], and all programming was implemented in Python using the scikit-learn libraries [39,40].

### 2.1 Feature extraction

**Fig 1:**
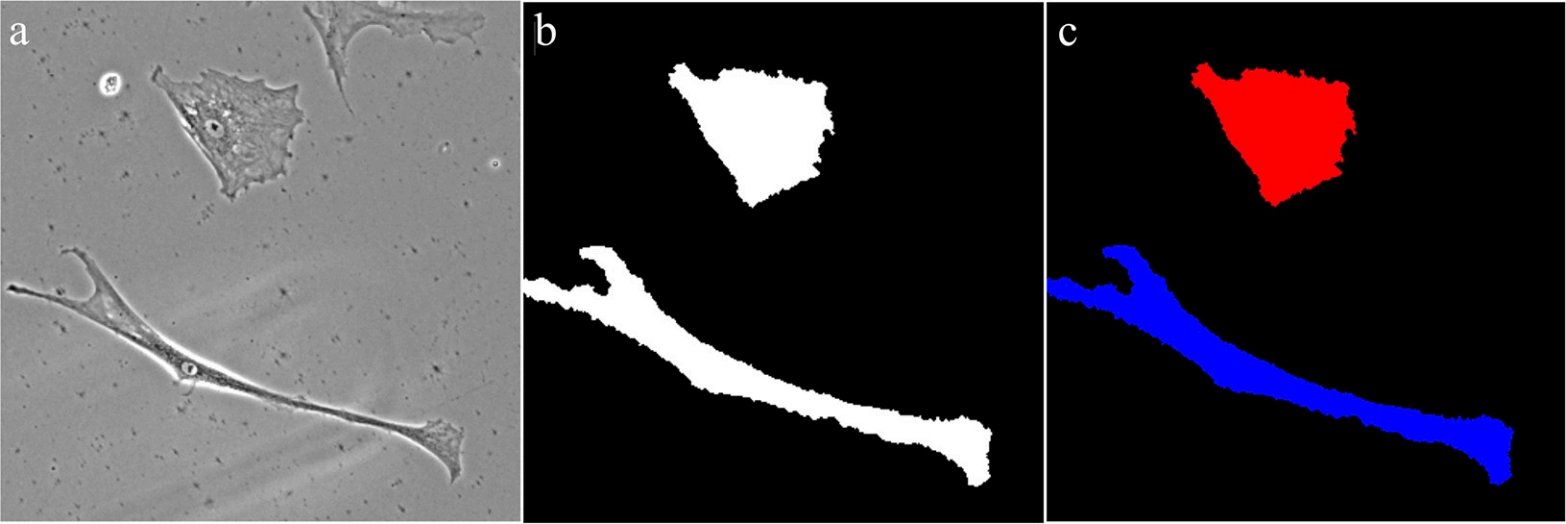
Process of feature extraction from images. **(a)** Phase contrast scans taken from a prior experiment in which HWJCs were differentiated into MHC-like cellss. (**b)** An empirical gradient threshold (EGT) was applied to the phase contrast scan to separate the cells from the background. **(c)** A minimum cross entropy thresholding (MCET) method was applied to the EGT images to identify cells and then size, shape, and intensity features were automatically extracted.

Prior to the development of the ML models, representative size, shape, and intensity features from each of the cells being used were extracted. To accomplish this task, several different procedures were employed, as shown in **Fig 1**. First, a total of 75 different phase contrast images were taken from an experiment in which HWJCs were differentiated into MHC-like cells [9]. An empirical gradient threshold (EGT) was applied to these phase contrast images in Fiji to automatically separate the foreground of the image from the background [41]. Afterwards, a minimum cross entropy thresholding (MCET) method was applied to the images in CellProfiler to accurately identify the outlines of the cells and extract size and shape features [42,43]. Intensity features of each cell were procured by overlaying cell outlines on the original phase contrast image. Data labels corresponding to treatment group and the number of days into the experiment that the image was taken were also included in the data set. After processing all 75 images, a total of 241 cells were identified, each with 33 features. For the purposes of this paper, overlapping cells or cells cut off by the edge of the picture were not used, because the extracted data for these cells were not representative of the actual cell. Using the extracted data, cells were manually classified as HWJC or MHC-like cells to complete the data set.

### 2.2 Determination of active features

Not all extracted features were necessarily relevant in regard to discerning MHC-like cells from HWJCs. Thus, a feature selection method was implemented to determine which features were pertinent to the classification of these cells. By filtering out certain features, the dimensionality of the data set used to develop ML models is reduced, decreasing the training time of the models and making the models less complex [44]. Moreover, feature selection methods often improve the performance of the models by filtering out information that may not be relevant to the classification problem [45]. In this paper, an *L*_*1*_ regularized LR was developed using all 241 cells, and the coefficient, or weight, of each feature was determined. Features with a nonzero weight were determined to be active features.

### 2.3 Development of machine learning models

Before the ML models were developed, the data set was randomly split into a training set of data and a testing set of data. The training set consisted of 80% of the original data, and the testing set contained the remaining 20% of the data. Each model was fit using the training data and then evaluated for performance on the testing set of data. After the data set was split into the training set and testing set, all features in both sets of data were normalized to ensure that one feature with a greater range did not intrinsically influence the models more than another feature [46]. Afterwards, each model was developed using the normalized training set of data. Any hyperparameters for each model were automatically tuned using a five-fold grid search cross-validation algorithm [47]. This method was used to tune the inverse of regularization strength (denoted as the parameter *C*) for the LR models [48], the penalty parameter (denoted as the parameter *C*) and kernel function for the SVM models [49], the number of neighbors used in the KNN algorithm [50], the split criterion for the random forest classifier [51], and the size of the hidden layer and regularization parameter (denoted as the parameter ⍺) for the MLP [52]. During the development of the VC model, the kernel function of the constituent SVM model and the split criterion of the random forest model were predefined to conserve computational resources and reduce the run time of the algorithm.

For each model, the entire process of development described here was done once using all features and once using only the active features. The performance of the algorithm using all features was compared to the performance of the algorithm using only the active features to determine if limiting the model to using only the active features had any effect.

### 2.4 Development of voting classifier

After all of the models were developed, performance metrics were obtained to determine which models had the best performances. A VC model was developed with the top performing models. Due to the amount of time and processing power that this model requires for development, the VC was only implemented using active features.

### 2.5 Performance evaluation

For each ML model, two different performance metrics were obtained for evaluation: the area under the curve (AUC) of the receiver operating characteristic (ROC) curve and an accuracy score. To compute the AUC, the ROC curve was constructed using the test data for each model. The accuracy score was calculated by comparing the predictions of each model on the test cases to their actual classes.

## 3. Results

### 3.1 Active features

**Fig 2:**
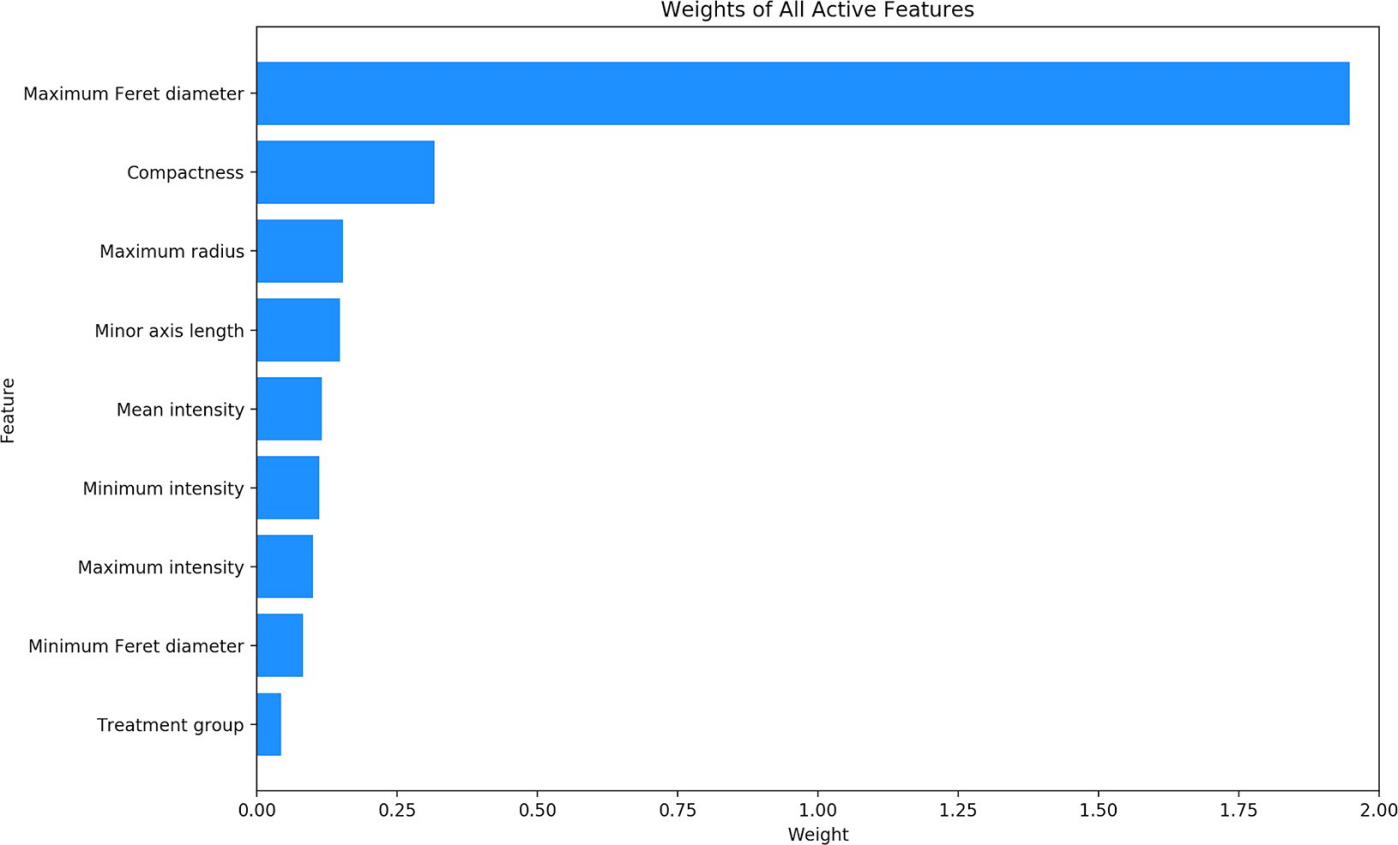
Weights of all active features. The absolute value of the weights of all active features, as determined by an *L*_*1*_ regularized LR.

After the process of feature extraction was completed, 33 features were extracted from each cell. Using an *L*_1_ regularized LR model on the complete set of data, nine of these 33 features were determined to be active features. The other 24 features were determined to be unrelated to classification. **T**he absolute value of the weight of each of the nine active features is illustrated in **Fig 2**. The features with the greatest weights were determined to be the maximum Feret diameter with a weight of 1.947, followed by compactness with a weight of 0.317, and maximum radius with a weight of 0.154. The exact weights of all nine active features can be found in **Table 1.**

**Table 1:** Weights of all active features.

Feature	Weight
Maximum Feret diameter	1.947
Compactness	0.317
Maximum radius	0.154
Minor axis length	0.148
Maximum intensity	0.101
Minimum Feret diameter	0.083
Treatment group	0.044
Minimum intensity	-0.112
Mean intensity	-0.116

The weights of all active features, as determined by an *L*_*1*_ regularized LR.

### 3.2 Variance of performance metrics

**Fig 3:**
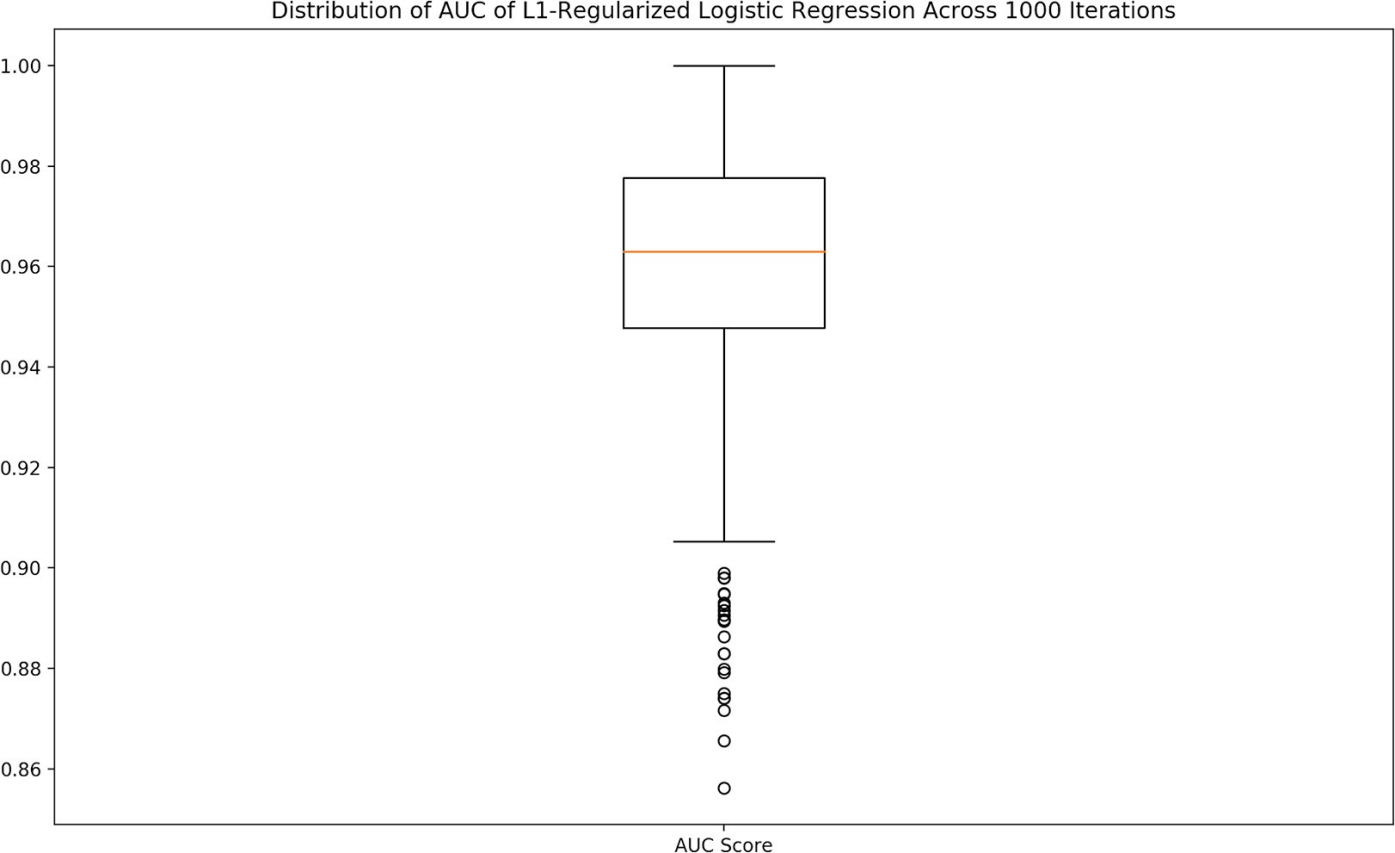
Variance of performance metrics. The performance metrics for each model could vary significantly due to the randomness of the split of the data into the training and testing set. To account for this variation, the median scores for each performance metric across 1,000 iterations was used. The median was chosen due to the fact that the data were significantly skewed.

It was observed that model performance could vary significantly across multiple runs due to the random split of the data into the training and testing sets. An example of this is illustrated in **Fig 3**, as the data show a wide range of possible AUC values for an *L*_*1*_ regularized LR across 1,000 runs. For accurate analysis and comparison, the entire process of development for each model was repeated 1,000 times, and the median AUC and accuracy across those 1,000 runs were used as the final performance metrics for that model.

### 3.3 Performance of machine learning models

The performance metrics of all six ML models implemented in this paper are listed in **Table 2**. Every model’s accuracy score was either 0.8571 or 0.8776 under any condition, indicating that a larger data set must be used to truly discern between the accuracies. The top performing algorithm was judged primarily based on the AUC, as it is more representative of the predictive capabilities of the model and accounts for the degree of confidence of each prediction made by the model [53]. The highest AUC of 0.9630 was achieved by both the *L*_*1*_ regularized LR and the *L*_*2*_ regularized LR when developed with active features, however all of the performance metrics were similar across the models.

**Table 2:** Performance metrics of models.

Algorithm	AUC score with all features	AUC score with active features (± increase over all features)	Accuracy score with all features	Accuracy score with active features (± increase over all features)
L1-regularized logistic regression	0.9490	0.9630 (+0.0140)	0.8571	0.8776 (+0.0205)
L2-regularized logistic regression	0.9483	0.9630 (+0.0147)	0.8776	0.8776 (+0.0000)
Support vector machine	0.9481	0.9615 (+0.0134)	0.8776	0.8776 (+0.0000)
Random forest	0.9582	0.9614 (+0.0032)	0.8571	0.8776 (+0.0205)
K-nearest neighbors	0.9352	0.9367 (+0.0015)	0.8571	0.8776 (+0.0205)
Multi-layer perceptron	0.9281	0.9298 (+0.0017)	0.8571	0.8571 (+0.0000)

Performance metrics of all six ML algorithms when developed with all features (columns 2 and 4) and when developed with only active features (columns 3 and 5).

### 3.4 Development of voting classifier

**Fig 4:**
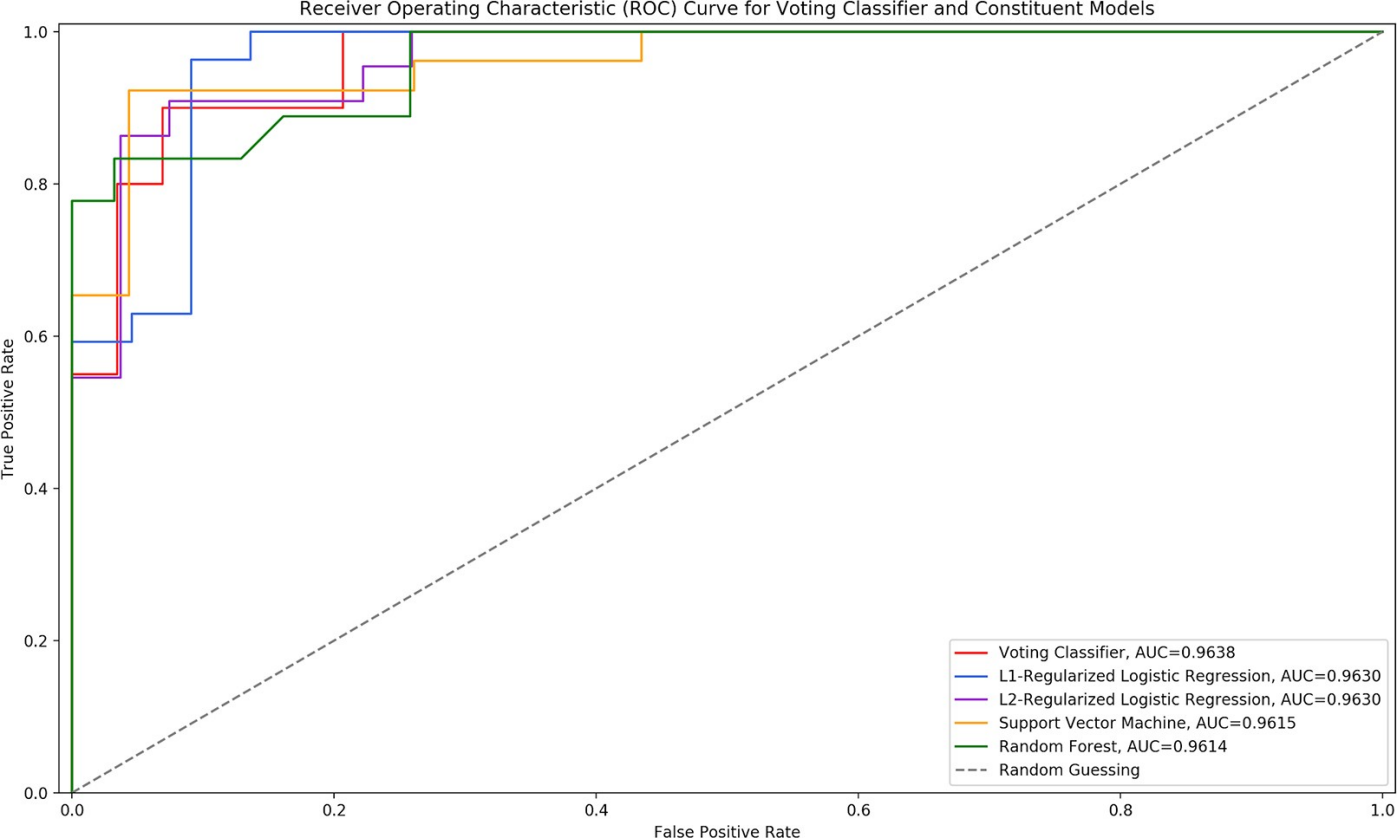
ROC curve of voting classifier and constituent models. The ROC curve of the voting classifier model as compared to its constituent models.

Using the results from **Table 2**, the top performing models were determined to be the *L*_*1*_ regularized LR, the *L*_*2*_ regularized LR, the SVM, and the random forest classifier. All of these models had very similar AUC scores when developed with active features, and all had equal accuracies. With these models, a VC model was developed. The ROC curves of the VC model and its constituent models can be seen in **Fig 4.** The performance of the VC model implemented in this paper as compared to its constituent algorithms can be seen in **Table 3.**

**Table 3:** Comparison of VC model with its constituent algorithms.

Algorithm	AUC score	Accuracy score
Voting classifier	0.9638	0.8980
L1-regularized logistic regression	0.9630	0.8776
L2-regularized logistic regression	0.9630	0.8776
Support vector machine	0.9615	0.8776
Random forest	0.9614	0.8776

All listed values are performance metrics based on the fit with active features only.

## 4. Discussion

### 4.1 Top performing model

The ROC curves of all models were analyzed using the MedCalc statistical software [54]. Each model’s ROC curve had *p*-value < 0.05, indicating a statistically significant difference between the model’s AUC score and an AUC of 0.50; thus, it can be concluded that each model had an ability to distinguish between HWJCs and MHC-like cells [54]. Moreover, the software was used to conduct a pairwise comparison of the ROC curves of the VC model and its constituent models. Each comparison had *p*-value > 0.05, revealing that there was no significant difference between the performance of the VC model and that of its constituent models. The VC model showed only a slight overall improvement in performance as compared to the other models, but in applications where thousands of cells are being identified, these slight differences may become more prominent. It was difficult to precisely rank the models presented in this paper, as many of the models had very similar performance metrics. For example, many of the accuracies obtained were equal across models. Larger data sets may help determine which models are more appropriate for this application.

### 4.2 Implications of limiting models to active features

An *L*_*1*_ regularized LR was implemented to identify active features, as this algorithm has the unique ability to set the weight of certain less important features to 0. Developing an *L*_*1*_ regularized LR with all 241 cells resulted in 9 of the 33 features having a nonzero weight. When training data was limited to these nine identified active features, performance metrics showed consistent improvement across almost all models. The 24 features that were removed likely carried redundant or irrelevant information, and as a result, removing them from the data set improved model performance. Ultimately, it can be concluded that the implemented feature selection method was successful in improving the performance metrics of the models.

### 4.3 Conclusion and future work

ML was applied in a novel way in this paper, and for the first time, individual models were combined to obtain a more accurate overall model for classifying HWJCs and MHC-like cells. The consistently high performance metrics of all models presented in this paper suggest that ML may be a viable alternative to manual classification in applications like this paper. Ultimately, the novel computational pipeline developed in this research may provide more utility in automating cell classification as compared to alternative methods that attempt to accomplish the same task. The computational pipeline presented in this paper has a feasible implementation and could potentially be generalized to other cell types in the future. Moreover, as compared to deep learning models, which can take days to train depending on the hardware available, the models developed in this paper have much lower training times and require far less computation resources, while still maintaining similar levels of performance [32]. In the future, the process of cell classification presented in this paper could be used in tissue engineering or gene therapy settings to automate cell classification or validate manual classification. Furthermore, the presented pipeline could potentially be adapted to not only classify MHC-like cells, but also determine their viability after differentiation. Ultimately, this pipeline could be tested on any cell classification problem where there is a change in cell size, shape, or texture between the cell populations.
